# 3D micropattern force triggers YAP nuclear entry by transport across nuclear pores and modulates stem cells paracrine

**DOI:** 10.1093/nsr/nwad165

**Published:** 2023-06-01

**Authors:** Yan Li, Zhenyu Zhong, Cunjing Xu, Xiaodan Wu, Jiaqi Li, Weiyong Tao, Jianglin Wang, Yingying Du, Shengmin Zhang

**Affiliations:** Advanced Biomaterials and Tissue Engineering Center, Huazhong University of Science and Technology, Wuhan430074, China; Department of Biomedical Engineering, Huazhong University of Science and Technology, Wuhan430074, China; Advanced Biomaterials and Tissue Engineering Center, Huazhong University of Science and Technology, Wuhan430074, China; Department of Biomedical Engineering, Huazhong University of Science and Technology, Wuhan430074, China; Advanced Biomaterials and Tissue Engineering Center, Huazhong University of Science and Technology, Wuhan430074, China; Department of Biomedical Engineering, Huazhong University of Science and Technology, Wuhan430074, China; Advanced Biomaterials and Tissue Engineering Center, Huazhong University of Science and Technology, Wuhan430074, China; Department of Biomedical Engineering, Huazhong University of Science and Technology, Wuhan430074, China; Advanced Biomaterials and Tissue Engineering Center, Huazhong University of Science and Technology, Wuhan430074, China; Department of Biomedical Engineering, Huazhong University of Science and Technology, Wuhan430074, China; Advanced Biomaterials and Tissue Engineering Center, Huazhong University of Science and Technology, Wuhan430074, China; Department of Biomedical Engineering, Huazhong University of Science and Technology, Wuhan430074, China; Advanced Biomaterials and Tissue Engineering Center, Huazhong University of Science and Technology, Wuhan430074, China; Department of Biomedical Engineering, Huazhong University of Science and Technology, Wuhan430074, China; Advanced Biomaterials and Tissue Engineering Center, Huazhong University of Science and Technology, Wuhan430074, China; Department of Biomedical Engineering, Huazhong University of Science and Technology, Wuhan430074, China; Research Base of Regulatory Science for Medical Devices, National Medical Products Administration & Institute of Regulatory Science for Medical Devices, Huazhong University of Science and Technology, Wuhan430074, China; Advanced Biomaterials and Tissue Engineering Center, Huazhong University of Science and Technology, Wuhan430074, China; Department of Biomedical Engineering, Huazhong University of Science and Technology, Wuhan430074, China; Research Base of Regulatory Science for Medical Devices, National Medical Products Administration & Institute of Regulatory Science for Medical Devices, Huazhong University of Science and Technology, Wuhan430074, China

**Keywords:** bioactive materials, tissue regeneration, 3D micropattern force, paracrine, mechanotransduction

## Abstract

Biophysical cues of the cellular microenvironment tremendously influence cell behavior by mechanotransduction. However, it is still unclear how cells sense and transduce the mechanical signals from 3D geometry to regulate cell function. Here, the mechanotransduction of human mesenchymal stem cells (MSCs) triggered by 3D micropatterns and its effect on the paracrine of MSCs are systematically investigated. Our findings show that 3D micropattern force could influence the spatial reorganization of the cytoskeleton, leading to different local forces which mediate nucleus alteration such as orientation, morphology, expression of Lamin A/C and chromatin condensation. Specifically, in the triangular prism and cuboid micropatterns, the ordered F-actin fibers are distributed over and fully transmit compressive forces to the nucleus, which results in nuclear flattening and stretching of nuclear pores, thus enhancing the nuclear import of YES-associated protein (YAP). Furthermore, the activation of YAP significantly enhances the paracrine of MSCs and upregulates the secretion of angiogenic growth factors. In contrast, the fewer compressive forces on the nucleus in cylinder and cube micropatterns cause less YAP entering the nucleus. The skin repair experiment provides the first *in vivo* evidence that enhanced MSCs paracrine by 3D geometry significantly promotes tissue regeneration. The current study contributes to understanding the in-depth mechanisms of mechanical signals affecting cell function and provides inspiration for innovative design of biomaterials.

## Teaser

This paper revealed a new mechanotransductive pathway of stem cells triggered by 3D micropattern force and its inducing effect on tissue regeneration, providing inspiration for innovative design of specific biomaterial surfaces.

## INTRODUCTION

Cells have the ability to sense and respond to biophysical properties of the extracellular matrix (ECM), such as matrix elasticity, rigidity and topography [1–3]. These biophysical cues mediate cell behaviors via mechanotransduction, a process that integrates and converts biophysical cues in the microenvironment to intracellular biochemical signals [4]. In general, these mechanical stimuli alter the formation of focal adhesions (FAs) and alignment of the cytoskeleton, initiating the signaling cascades that activate transcription factors [5]. In addition, growing evidence shows that the cell nucleus is directly or indirectly submitted to a force that can act as a mechanosensor [6]. The forces transmitted to cells, specifically the nucleus, affect the nucleocytoplasmic localization of specific transcriptional regulators involved in different signalling pathways, such as MRTF-A, β-catenin or YAP, which further regulate gene expression to control cellular phenotypes [7–9]. For example, the mechanical properties of the cellular microenvironment reveal the fundamental effects on human mesenchymal stem cells (MSCs) function and tissue regeneration [10,11].

Among these mechanical cues, the micropatterns with various geometries have shown to significantly tune cell behaviors [12]. 2D micropatterned substrates have been used to investigate the correlation between cell shape and cell viability. Reduced micropattern area has been shown to result in decreased cell proliferation and increased cell death [13,14]. Studies have verified that the geometries can affect the organization of stress fibers and FAs in 2D substrates [15]. Furthermore, the aggregation of tensional stress in the corner areas of rectangular islands can improve lamellipodia contractility [16]. In addition, the stem cells on adhesive islands of multifarious geometries but with invariable area have the diverse potential of cell differentiation [17]. Dextral geometry enhanced osteogenic differentiation of MSCs compared with sinistral geometry [18]. Current studies have demonstrated that rather than stem cell differentiation, the paracrine function of MSCs plays a vital role in their therapeutic benefits for tissue regeneration, however, the knowledge of how geometry affects MSCs paracrine is still lacking [19–21].

Despite extensive studies on 2D substrate geometry to direct cell behavior, they do not fully recapitulate the critical characteristics of the native 3D cell niche. Stem cells reside in a complex 3D microenvironment *in vivo*, where the multidimensional stimuli integrate to control a series of cell fates involving cell survival, self-renewal, differentiation and paracrine [22,23]. It is instructive to understand how mechanosensing of stem cells works in the 3D niche, as it would lead to a much better understanding of how cells develop and enhance their distinctive function, and thus provide the guidance for the material design to remodel tissue regeneration. Recent studies have investigated the extent to which the confinement of 3D microniches impact actin polymerization, cell viability and differentiation [24,25]. However, it remains unknown how stem cells sense mechanical signals from 3D geometry and subsequently transmit them to the nucleus to regulate cell paracrine function.

In this work, we constructed well-defined 3D micropattern arrays with different curvature shapes (cylinder, triangular prism) and diverse aspect ratios (cubic, cuboid) to reveal the mechanotransduction of MSCs triggered by 3D geometry and its effect on the paracrine of MSCs. The 3D micropatterns enable control of the individual cell adhesion and spread to the constrained geometry shape. The effect of 3D micropatterns on the spatial reorganization of FAs, orientation and tension of stress fibers, and their mechanical connection to the nucleus have been systematically explored. Our findings showed that the triangular prism and cuboid micropatterns influenced the orientation of F-actin and stretch of nuclear pores, further enhancing the nuclear import of YAP, which significantly enhanced the angiogenic paracrine responses of MSCs, resulting in enhanced vascularization and wound restoration in a rat model.

## RESULTS AND DISCUSSION

### Fabrication of 3D micropattern arrays and single MSCs occupancy in the microwells

**Figure 1.**
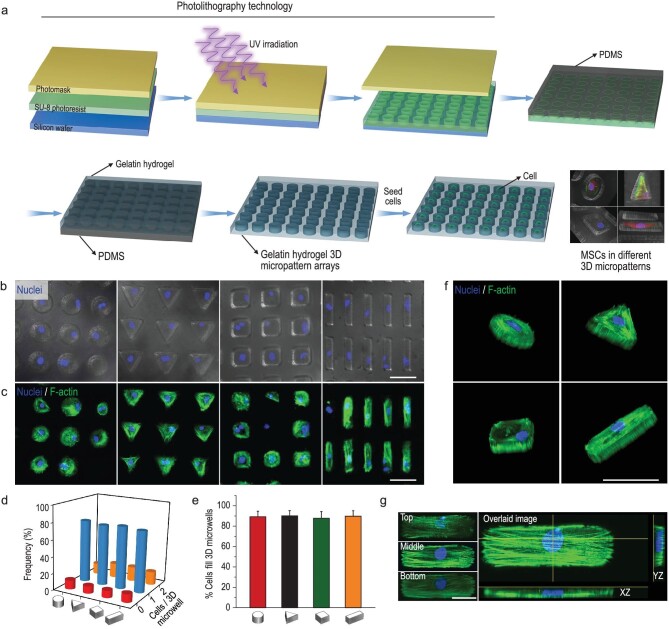
Fabrication of 3D micropattern arrays and single MSCs occupancy in the microwells. (a) Schematic illustration of the process for fabricating hydrogel 3D micropattern arrays. (b) Fluorescence images showed nuclear staining of the single cell encapsulated in 3D micropatterns with different geometries but the same volume (cylinder, triangular prism, cubic, and cuboid). Scale bar, 50 μm. (c) Confocal images showed that MSCs morphology was well controlled by the 3D micropattern. The F-actin and nucleus were labeled with phalloidin (green) and DAPI (blue), respectively. Scale bar, 50 μm. (d) Quantitative results of MSCs encapsulation efficiency in the 3D micropattern with different geometries. (e) Quantitative results of cells filling in 3D microwells with different geometries; the regions for quantitative analysis were selected randomly, *n* ≥ 4. (f) Three-dimensional reconstruction of F-actin and nucleus in different 3D micropatterns, green: F-actin, blue: nucleus. Scale bar, 50 μm. (g) The overlaid confocal image showed that F-actin was well arranged in cuboid micropatterns. Overlaid image was generated by merging multi–z-stack images into a single stack. Scale bar, 20 μm.

We manufactured gelatin hydrogel 3D microwell arrays that contained the controlled geometry and volume through lithography techniques and microfabrication (Fig. 1a). To explore the influence of 3D geometry on MSCs function, we generated 3D micropattern arrays with different curvature shapes (cylinder and triangular prism) and aspect ratios (cubic and cuboid), with the same project area of 1256 μm^2^ and height of 40 μm (see Supplementary Fig. S1 in the online supplementary file).

After the preparation of 3D microwell arrays, MSCs were sedimented into the microwells by seeding a suspension of cells on the top of the patterned hydrogel surface. Once the MSCs deposited into the microwells, the excess cells were removed from the platform surface by washing with culture medium. The MSCs numbers were counted by nucleus staining to determine the individual cell occupancy efficiency of 3D microwells. The staining results proved that the modified 3D micropatterns possessed excellent individual MSC occupancy efficiency (Fig. 1b and c). The quantitative results showed that over 75% of microwells were occupied by a single cell (Fig. 1d). The MSCs spreading process in the microwells was recorded during 24 h culturing (Supplementary Fig. S2). The cell obtained a spread shape after 5 h, and remained stable during the subsequent imaging experiment. Figure 1c and e showed that single cells spread well in the microwells and grew into the shape of the micropatterned geometry by up to 90%. The 3D reconstruction images further showed that the cytoskeleton of the single cell was completely confined by 3D micropatterns (Fig. 1f). To clearly visualize 3D micropatterned MSCs, the confocal images from different z-stacks were acquired and then merged into an overlaid image. As shown in Fig. 1g, F-actin formed arches above the nucleus at the two ends along the principal axis of the cells. The overlaid image also demonstrated that the micropatterns fabricated with our method could well confine the cytoskeleton (Fig. 1g). Taken together, we have manufactured different 3D micropattern arrays with high individual MSC occupancy efficiency (over 75%) and favorable cell shape confinement capability, which provide support for subsequent studies on the mechanism of mechanical signaling transduction induced by 3D geometry.

### 3D micropatterns affect single-cell contractility by inducing spatial reorganization of focal adhesions and cytoskeleton

**Figure 2.**
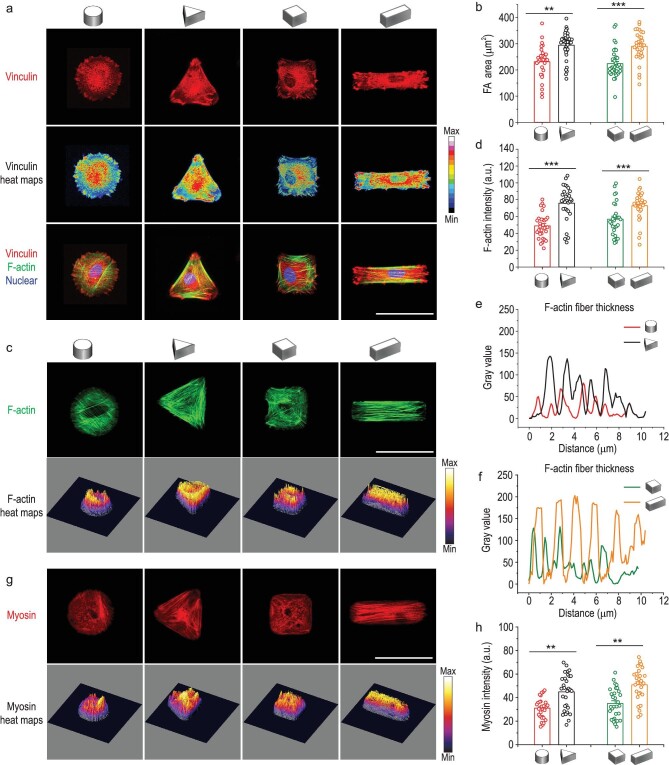
Spatial reorganization of focal adhesions and cytoskeletal in response to cell shape changes in 3D micropatterns. (a) Representative confocal images of vinculin (red) identifying focal adhesions, F-actin (green) and nucleus (blue) for the single cell in 3D micropatterns. Scale bar, 50 μm. (b) Quantification of the total focal adhesions area of cells confined in 3D micropatterns. (c) Fluorescence images of F-actin filament staining for MSCs cultured in 3D micropatterns. Scale bar, 50 μm. (d) Quantification of fluorescence intensity of F-actin in different 3D micropatterns. (e and f) F-actin fibers thickness in 3D micropatterns evaluated by fluorescence intensity. (g) Representative fluorescence images of myosin staining of MSCs cultured in 3D micropatterns. Scale bar, 50 μm. (h) Quantification of myosin intensity of MSCs cultured in 3D micropatterns. The data are represented as the mean ± SD, *n* = 30–40 cells per condition, ** *p* < 0.01, *** *p* < 0.001.

The mechanical characteristics of the extracellular matrix (ECM) can be perceived through focal adhesions (FAs), which transform the physical cues into intracellular biochemical signals that further regulate downstream signaling pathways [26]. Thus, FAs play an important role in mediating the attachment and anchorage of cells to the ECM. Studies have shown that the size of FAs could directly reflect the tension level of cells [27]. When the FAs are large, the anchorage is firm, and the cell is in a state of strong mechanical tension. Therefore, we focused on the FAs spatial assembly after MSCs being confined in 3D micropatterns with different curvature shapes (cylinder and triangular prism) and aspect ratios (cubic and cuboid). As shown in Fig. 2a, FAs of MSCs were observed in all 3D micropatterns, while larger FAs were expressed in the curvature regions of the triangular prism compared with the cylinder and at the long axis edge of the cuboid compared with cube, indicating firm anchorage of MSCs in both the triangular prism and cuboid patterns. As shown in the merged fluorescence images, large FAs mainly appeared at the ends of F-actin stress filaments. The quantification of FAs displayed that the total area of FAs was significantly higher in triangular prism and cuboid groups compared to the other two groups (Fig. 2b). Our findings showed that the spatial organization and extent of FAs were significantly influenced by 3D micropattern parameters including curvature shape and aspect ratio. Specifically, cellular tension would increase with sharper curvature and higher aspect ratio of the pattern.

It has been reported that FAs and cytoskeleton are involved in regulating the cellular mechanical response to ECM signals [28]. In addition, the actomyosin cytoskeleton has been shown to play critical roles in mechanosensing and mechanotransduction processes [29]. The F-actin and myosin of MSCs were fluorescence stained to examine the effect of 3D micropatterns on cytoskeleton organization (Fig. 2c and g). F-actin staining showed different stress fiber organization, with more stress fibers observed in triangular prism and cuboid patterned cells. Quantification of F-actin fluorescence revealed significantly higher levels of F-actin in cells with triangular prism and cuboid geometry compared to cells in cylindrical and cubical micropatterns (Fig. 2d). Similarly, the thickness of F-actin stress fibers increased with the increase of cells geometry curvature or aspect ratio (Fig. 2e and f, and Supplementary Fig. S3). This suggested that the cytoskeleton was strengthened when MSCs were confined in the triangular prism or cuboid micropattern. The thickness of stress fibers is positively correlated with F-actin tension, because stress fibers are composed of F-actin bundles that are cross-linked by various actin-binding proteins [30]. The amount and activity of these cross-linking proteins can be regulated by the mechanical tension of F-actin, resulting in changes in stress fiber thickness. Studies have shown that increased mechanical tension on F-actin leads to enhanced recruitment and activity of stress fiber cross-linking proteins, such as α-actinin and filamin [31].

Moreover, MSCs with the triangular prism and cuboid geometry demonstrated 140% and 145% higher intensity signals for myosin compared with MSCs cultured in cylindrical and cubical microwells, respectively (Fig. 2h). It implied that the expressed level of myosin was also dependent on cellular curvature shape or aspect ratio. The bound myosin has been reported to generate traction force along the F-actin stress filaments, and thereby affecting cell mechanics [24]. Consequently, we confirmed that the 3D micropatterned MSCs with increased curvature shape or aspect ratio underwent an enhanced force-dependent mechanism through FAs and spatial reorganization of the actomyosin cytoskeleton.

### 3D micropatterns regulate nuclear shape remodeling and nuclear mechanics

The cell nucleus where transcription takes place is another important part of mechanotransduction that affects the cellular mechanical properties and bioactivities [7]. Following adhesion, the cytoskeleton reassembles to transmit signals from the ECM to the nucleus, triggering multifarious cellular events [32,33]. While the nuclear morphology in 3D micropatterned MSCs and its correlation with F-actin filaments are still a mystery, we therefore focused on the response of nucleus morphology and mechanics to 3D micropatterns.

**Figure 3.**
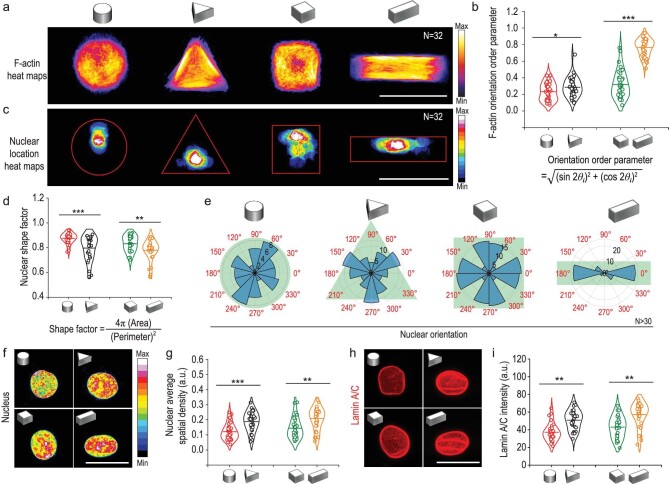
3D geometric constraints regulate cellular F-actin orientation, nuclear morphology and mechanics. (a) F-actin immunofluorescence intensity heat maps of MSCs cultured in 3D micropatterns. N was the number of cells used for heatmap generation. Scale bar, 50 μm. (b) F-actin orientation order parameter in 3D micropatterned cells. (c) Nucleus location heat maps of cells cultured in 3D micropatterns. (d) Quantification of nuclear shape factor estimated from projected nuclear morphology. (e) Angular graphs, superimposed on micropattern drawings in reseda, showed the different orientations of nuclei in response to different 3D micropatterns. Here, N was the number of cells used for nucleus orientation analysis. (f and g) Chromatin condensation was visualized by generating a heatmap of the DAPI intensity. Quantitation of nucleus average spatial density (overall fluorescence intensity per nuclear volume) for 3D micropatterned cells. Scale bar, 20 μm. (h and i) Representative confocal images of nucleus Lamin A/C and quantitation of nucleus Lamin A/C intensity level of MSCs cultured in 3D micropatterns. Scale bar, 20 μm. The data were represented as the mean ± SD, *n* = 30–40 cells per condition, * *p* < 0.05, ** *p* < 0.01, *** *p* < 0.001.

Figure 3a and c showed the spatial distribution of nucleus position in MSCs with different geometries. Note that the nucleus was located further from the pattern boundary in cylinder than that in triangular prism microwell, while the nucleus site was closer to the long axis of cuboid pattern with higher aspect ratio compared with cube pattern. The F-actin arrangement of MSCs in 3D micropatterns was further assessed quantitatively according to a previous report [34]. The F-actin orientation order parameter = $\sqrt {{{\rm (sin2{\theta }_{\rm{i}})}}^2 + {{(\cos 2{\theta }_{\rm{i}})}}^2} $ increases from 0 for randomly oriented features up to 1 for perfectly aligned features. The statistical results suggested that the F-actin arrangement tended to be more aligned as MSCs curvature angles or aspect ratios increased (Fig. 3b). The nuclei shape factor was calculated based on the projection of nuclear morphology on the XY-plane (Fig. 3d). It revealed that the nucleus in triangular prism and cuboid micropatterns exhibited an elongated shape compared with those in cylinder and cubic groups, respectively (Fig. 3d). It could be concluded from Fig. 3b and d that the shape of the nucleus became more elongated when the F-actin orientation order increased. Next we studied the correlation between 3D geometry and nuclear orientation. Nuclear orientation of MSCs in cylindrical micropatterns showed a random assignment spanning 360°. However triangular prism-patterned MSCs had a preferential nucleus orientation toward three boundary sides (Fig. 3e). As micropatterns aspect ratio increased, the F-actin orientation order parameter increased, and nuclei became a well-oriented state aligned with the long axis of cuboid micropatterns (Fig. 3e).

Nuclear mechanics regulated by substrate stiffness is associated with chromatin remodeling, which impacts a sequence of essential cellular processes, such as mRNA transcription, secretome, repair and so on [6,35]. We investigated chromatin condensation by creating heatmaps based on DAPI staining (Fig. 3f). Indeed, the uptake of DAPI depends on the entire amount of DNA and its level of condensation. The higher average nuclear spatial density in triangular prism and cuboid patterned MSCs showed a higher gene clustering level compared with those in cylinder and cube groups (Fig. 3f and g). This indicated that chromatin remodeling in MSCs was regulated by cell mechanics that triggered by 3D micropatterns. As an intermediate filament protein, Lamin A/C exists in almost all cells and plays an important role in nucleus-cytoskeleton junction-dependent mechanotransduction [36]. Therefore, the Lamin A/C is usually regarded as a sensor for nuclear mechanics [37]. We then investigated the mechanical reply of the nucleus to 3D micropatterned physical signals with fluorescence images of Lamin A/C (Fig. 3h). The MSCs in triangular prism and cuboid patterns showed higher expression of Lamin A/C and a grooved surface of nuclear membrane. Quantitative results of Lamin A/C intensity displayed 1.4 times higher Lamin A/C expression levels in MSCs with triangular prism geometry compared to those with cylinder geometry. The Lamin A/C intensity was 1.6 times higher in MSCs with cuboid geometry compared to those with cube geometry (Fig. 3i). These results indicated that greater intracellular tensions were imposed on the nucleus of MSCs in triangular prism and cuboid patterns. It was reported that increased Lamin A/C levels were found in stiff tissues and in cells cultured on stiff artificial substrates [38]. Therefore, our findings showed that 3D geometry was a further parameter that could regulate the orientation order of stress fiber, nucleus remodeling and associated changes in Lamin A/C expression.

### Nuclear deformations reduce the mechanical restriction of nuclear pores on YAP translocation

**Figure 4.**
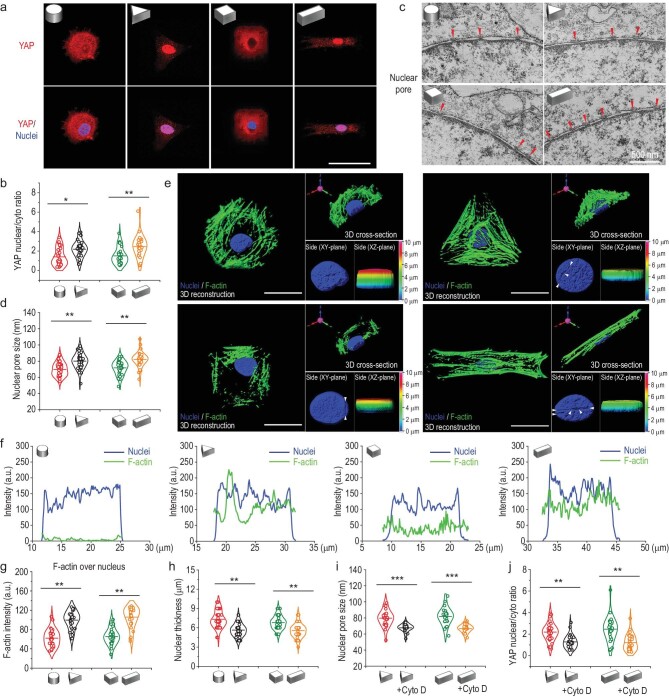
Nuclear deformations decrease the mechanical restriction of nuclear pores on YAP translocation. (a) Confocal microscopy images of YAP (red) in MSCs cultured in 3D micropatterns. Scale bar, 50 μm. (b) Quantification of nuclear/cytosolic YAP ratios in MSCs cultured in 3D micropatterns; *n* = 30–40 cells per condition. (c) TEM images of nucleus pores in MSCs cultured in 3D micropatterns. The small red triangle indicates the position of the nuclear pore. (d) Quantification of nuclear pore size in MSCs cultured in 3D micropatterns; *n* ≥ 40 nuclear pores from ≥ 15 cells per condition. (e) Representative cell and nuclear morphology in 3D micropatterns were captured by 3D reconstruction of fluorescence confocal images along the z-axis. Note that side views of pseudocolored 3D z-depth rendered nuclei display the thickness of nucleus. Scale bars, 20 μm. (f) The intensity plot of F-actin fibers and the nucleus of the micropatterned cells 3D cross-section. (g) Quantification of F-actin intensity that overlaps with nucleus in a 3D microniche with various geometries. Individual points and means are shown; *n* = 30–40 cells per condition. (h) Quantification of nucleus thickness in a 3D microniche with various geometries; *n* = 30–40 cells per condition. (i) Effects of F-actin inhibitor on nuclear pore size in micropatterned MSCs; *n* ≥ 40 nuclear pores from ≥ 15 cells per condition. (j) Effects of F-actin inhibitor on nuclear/cytosolic YAP ratios in micropatterned MSCs; *n* = 30–40 cells per condition. The data are represented as the mean ± SD, * *p* < 0.05, ** *p* < 0.01, *** *p* < 0.001.

Yes-associated protein (YAP) is a mechanosensitive transcriptional regulator that plays a critical role in regeneration, development, differentiation and organ size control [39]. YAP has been reported to be a master regulator of mechanotransduction, and its functions rely on the translocation from cytoplasm to nucleus [8,40]. Therefore, the nuclear translocation of YAP in MSCs cultured in 3D micropatterns was investigated to determine the behavior of YAP in response to different geometries. The level of YAP nuclear accumulation was noticeably higher in triangular prism and cuboid patterned MSCs compared to that in cylindrical and cubical patterns, which was consistent with the stress fiber results above (Fig. 4a and b). How mechanical signals induced by 3D micropatterns regulate translocation of YAP into nucleus remains unknown.

Further, the mechanism by which 3D micropattern-induced mechanical signals regulate YAP translocation into nucleus was investigated. 3D reconstruction results showed that F-actin fibers were distributed throughout the whole nucleus in triangular prism and cuboid patterned MSCs while few F-actin fibers were observed overlapping with the nucleus in cylindrical and cubic patterned MSCs (Fig. 4e). Intensity plot and quantification results of F-actin fibers over nucleus further proved this tendency (Fig. 4f and g) This discrepancy may be due to 3D geometrical constraints inducing alterations of F-actin level, F-actin orientation, nuclear orientation and nuclear location. In triangular prism and cuboid micropatterns, the stress fibers overlapping the nucleus exert a strong force on the nucleus, leading to nuclear flattening (Fig. 4e and h). The small white triangles in Fig. 4e indicate the indentations caused by strong pressure of F-actin on the nuclear envelope, resulting in changes in nuclear morphology. In addition, there was no significant difference in nuclear volume among the different 3D micropatterns (Supplementary Fig. S4). Therefore, it is reasonable to assume that the nucleus was under vertical pressure from the stress fibers above. All the results implied that the nucleus of MSCs with triangular prism and cuboid geometry acted under stronger force exerted by F-actin fibers compared with those in cylindrical and cubic patterns.

Nuclear pore complexes regulate nucleo-cytoplasmic transport, controlling the nuclear concentration of several transcription factors [41]. Because the nuclear pore lumen is composed of a flexible, disorganized protein meshwork containing phenylalanine-glycine repeats which impairs free diffusion and exerts mechanical resistance, the nuclear flattening may increase nuclear pore permeability [42]. Thus, nuclear pores in micropatterned MSCs were investigated with transmission electron microscopy (TEM). The size of nucleus pores was considerably larger in triangular prism and cuboid patterned MSCs than that in cylindrical and cubic micropatterns (Fig. 4c and d). To further prove that the increased nuclear pore size was due to F-actin contractility on the nucleus, MSCs in triangular prism and cuboid micropatterns were treated with cytochalasin D, an F-actin stress fiber polymerization inhibitor. It was found that after the treatment, the size of nuclear pores in MSCs was significantly reduced (Fig. 4i and Supplementary Fig. S6), and YAP nuclear localization was decreased (Fig. 4j). These results suggested that the pressing force onto the nucleus from the F-actin was sufficient to increase the nuclear entry of YAP by nuclear pore stretching. The mechanical mechanism of YAP nuclear translocation in triangular prism and cuboid micropatterns was the pressing force of F-actin on the nucleus decreasing the mechanical restriction of nuclear pores to molecular transport, thus enhancing the nuclear import of YAP. Our findings revealed a mechanosensing mechanism ultimately mediated by nuclear pores, demonstrated for YAP but with potential general applicability in nucleocytoplasmic shuttling of other proteins. This may contribute, for instance, to the localization of other mechanosensitive transcriptional proteins such as MRTF-A or β-catenin [43,44]. This mechanism may be central to influence long-term gene expression in response to mechanical cues, placing force transmission to the nucleus as a fundamental factor.

### 3D micropattern mechanical force regulates the paracrine function of MSCs and the underlying molecular mechanisms

**Figure 5.**
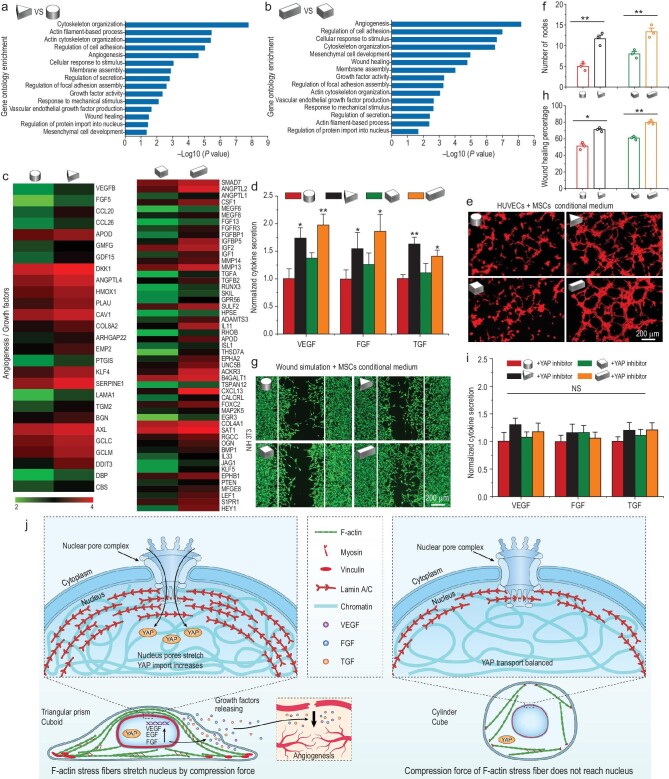
3D micropattern mechanical force regulates MSCs paracrine function by YAP nuclear localization. (a and b) Gene ontology analysis of significantly regulated genome in 3D micropatterned cells. (c) Gene chip heatmaps for the differential genes related to angiogenic factor and growth factor in 3D micropatterned cells. (d) ELISA measurement of the paracrine products of pro-angiogenic and pro-regenerative factors secreted by MSCs cultured in 3D micropatterns. (e) Tube formation images of HUVECS incubated with paracrine products from different 3D micropatterned MSCs for 6 h. (f) Quantitative analysis of tube formation assay. (g) Representative images of NIH 3T3 migration to the defined ‘artificial wound’ after treatment with conditional medium from different 3D micropatterned MSCs for 24 h. (h) Quantification of wound healing percent on NIH 3T3 cells by wound simulation scratch assay. (i) Effects of YAP inhibitor on the secretion of pro-angiogenic and pro-regenerative factors of 3D micropatterned MSCs detected by ELISA measurement analysis. The data are represented as the mean ± SD, * *p* < 0.05, ** *p* < 0.01. NS, no significant difference. (j) Schematic representation of mechanotransduction pathways of MSCs triggered by 3D micropatterns and its effect on paracrine function of MSCs.

The cellular function can be impacted by signals from the surrounding microenvironment [45]. For instance, numerous studies have demonstrated that MSC differentiation and tissue regeneration are significantly influenced by the mechanical properties of the cellular microenvironment, such as matrix stiffness, topographic features and surface curvature [46,47]. Although geometry has been demonstrated to regulate MSCs differentiation, few studies have examined how geometry affects MSCs paracrine function, which plays a pivotal role in tissue regeneration [48]. Through the paracrine pathway, MSCs secrete all kinds of trophic factors that actively respond to exterior environmental cues and mediate the immunomodulation, angiogenesis as well as tissue regeneration processes [49,50]. In this section, gene chip analysis was performed to explore the effects of 3D geometry on the paracrine function of MSCs. A differential gene expression analysis showed that different 3D geometry confinement strongly regulated numerous genes of MSCs (Supplementary Fig. S6). Gene ontology enrichment analysis revealed the differentially enriched gene groups, including angiogenesis, growth factor activity, vascular endothelial growth factor production, regulation of secretion and wound healing in triangular prism patterned MSCs compared to cylindrical patterned MSCs (Fig. 5a). Similar results were also shown in cuboid and cube groups (Fig. 5b). Moreover, the gene enriched in regulation of focal adhesion assembly, cytoskeleton organization, response to mechanical stimulus and regulation of protein import into nucleus further proved our above results that 3D micropatterns could exert various mechanical signals on MSCs and induce different cell behaviors (Fig. 5a and b). Detailed gene expression about angiogenesis and cell growth were further investigated by gene heat map analysis (Fig. 5c). Many pro-angiogenic and pro-regenerative genes were more highly expressed in triangular prism and cuboid patterned MSCs compared with cylindrical and cubic patterned MSCs, respectively (Fig. 5c).

Then, the mRNA and protein expression level of several typical pro-angiogenic and pro-growth factors in MSCs cultured in 3D micropatterns were examined by real-time reverse transcription-polymerase chain reaction (RT-PCR) and enzyme-linked immunosorbent assay (ELISA) analysis, respectively. The vascular endothelial growth factor (VEGF), fibroblast growth factor (FGF) and transforming growth factor (TGF) play important roles in regulating intracellular signal transduction and promoting cell proliferation, migration, differentiation and angiogenesis [19,21]. The gene expression of these growth factors was prominently increased in MSCs in triangular prism and cuboid micropatterns (Supplementary Fig. S8). Further, the protein secretion quantified by ELISA presented similar results to gene expression, which implied the triangular prism and cuboid micropatterns owned a better inducing effect on MSCs to secrete pro-regenerative cytokines (Fig. 5d).

Angiogenesis is considered a vital step for tissue regeneration, and there is a close cross-communication between HUVECS (Human Umbilical Vein Endothelial Cells) and MSCs. Subsequently, tube formation assay of HUVECs was applied to further verify the pro-angiogenesis ability of paracrine factors secreted by micropatterned MSCs. As shown in Fig. 5e, the conditioned medium (CM) from the triangular prism and cuboid patterned MSCs had the ability to promote microtube formation compared with the other groups, which may be due to the angiogenic effect of paracrine factors secreted by MSCs and accumulated in the media. The quantitative results showed that tube numbers were increased by 2.33-fold in triangular prism-CM compared to cylinder-CM. It also showed a 1.67-fold increase in cuboid-CM group compared with cube-CM group. These results strongly proved the potent pro-angiogenesis ability of paracrine products derived from triangular prism and cuboid groups. NIH-3T3 (mouse embryo fibroblasts) are an important cell type in skin tissue and play a crucial role in the synthesis of collagen [51]. In addition, a favorable migration of fibroblasts induces a fast re-epithelialization at the wound site, which is essential for skin repair [52]. Therefore, NIH-3T3 were used for scratch assay in this study, which reflected the migration ability of fibroblasts. The migration results of NIH-3T3 verified that paracrine factors from the triangular prism and cuboid-CM groups showed better pro-migration ability compared to the cylinder and cube-CM groups (Fig. 5g and h).

Next, we explored the underlying mechanism by which the 3D micropatterns regulated the paracrine profiles of MSCs. We focused on the YAP pathways, one of the most prominent pathways for ECM cues mechanotransduction. YAP localization was strongly dependent on the geometry of 3D micropatterns. The triangular prism and cuboid micropatterns significantly enhanced the activation of YAP compared to the cylindrical and cubical micropatterns. To investigate the relationship between the mechanotransduction and paracrine function of MSCs triggered by 3D micropatterns, the inhibitor verteporfin was used to eliminate the function of YAP. The protein secretion levels of paracrine factors VEGF, FGF, TGF were substantially downregulated when YAP was inhibited (Fig. 5i). Combining these results, the schematic representation in Fig. 5j summarizes the mechanical signals transduction mechanism of MSCs triggered by 3D micropatterns and its effect on paracrine function of MSCs. 3D micropatterns trigger the spatial reorganization of cytoskeleton, leading to different local forces, which mediate important alterations of the nucleus including orientation, morphology, expression of Lamin A/C and chromatin condensation. Specifically, in the triangular prism and cuboid micropatterns, ordered F-actin fibers distribute over the nucleus and fully transmit compressive forces to the nucleus and result in stretching of nuclear pores, thus enhancing the nuclear import of YAP. Furthermore, the activation of YAP enhances the angiogenic paracrine responses of MSCs.

### Paracrine products of 3D micropatterned MSCs affect angiogenesis and skin wound regeneration *in vivo*

**Figure 6.**
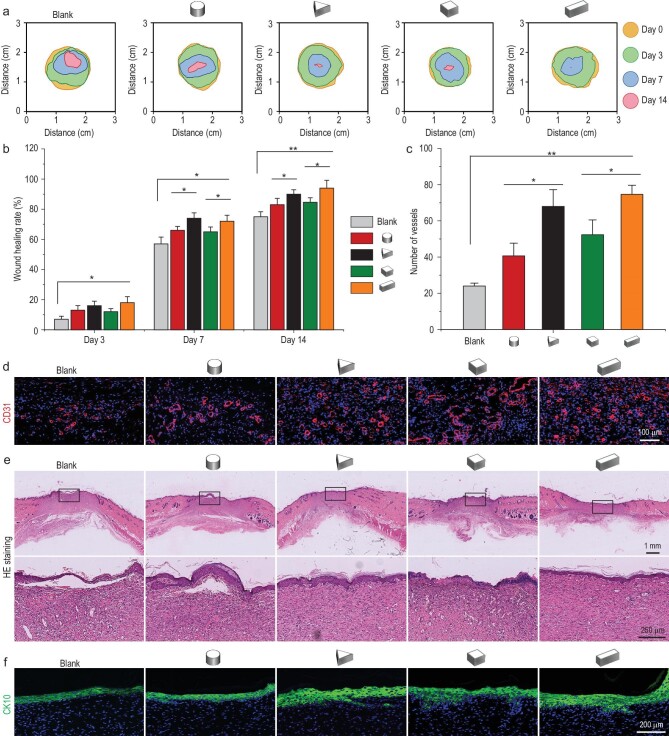
*In vivo* performance of the paracrine products of 3D micropatterned MSCs on skin wound regeneration. (a) Traces of wound-bed closure during 14 days (day 0, 3, 7 and 14) for different groups based on rat full-thickness cutaneous wounds. (b) Wound healing rates for each group on day 3, 7 and 14. (c and d) Quantification and representative immunofluorescent images of neovascularization for each group on day 7. (e) Skin regeneration in the wound site was assessed by H&E staining for each group on day 14. (f) Immunofluorescent images of CK-10 indicated the epithelial reconstruction for each group. The data are represented as the mean ± SD, * *p* < 0.05, ** *p* < 0.01.

A rat full-thickness skin wound model was established to further validate the effects of paracrine products secreted from MSCs cultured in different 3D micropatterns in wound regeneration. The contour of the wound bed from each group was drawn and superimposed for all groups, and the wound area gradually decreased as healing progressed (Fig. 6a). Particularly, the triangular prism and cuboid groups performed optimally in the acceleration of wound repair over the whole period (Fig. 6a and b, and Supplementary Fig. S9). This was consistent with the *in vitro* result that growth factors produced by the triangular prism or cuboid micropatterned MSCs accelerated the migration of fibroblasts, one of the key stages in wound healing. For wound healing, revascularization is also a crucial factor [11,20]. The vascular density was observed and quantified by CD31 (a marker of newly formed blood vessels) immunofluorescence staining. On day 7, angiogenesis was enhanced in wounds treated with paracrine factors of triangular prism or cuboid-CM groups compared to the other groups, respectively (Fig. 6c and d), which is consistent with the *in vitro* result of tube formation assay.

To evaluate whether the specific shapes of micropatterned MSCs could promote the regeneration of skin structures by secreted growth factors, HE (hematoxylin & eosin) staining and CK10 (marker of keratinocytes) immunofluorescent staining were performed. On day 14, a completely regenerated epithelium tissue was formed in triangular prism and cuboid groups, suggesting that the healing was almost complete (Fig. 6e). The enlarged part of the blank group displayed no histological connection between the regenerated epithelium and subcutaneous granulation tissue. The epithelium tissue in the cylinder and cube groups had not been fully regenerated (Fig. 6e). In 3D micropattern groups, the triangular prism and cuboid groups displayed stronger green color for CK-10 expression than the other groups, indicating a higher degree of keratinization and structural integrity of regenerated tissue (Fig. 6f; see online supplementary material for a color version of this figure). The expression of CK-10 in the blank group was least showing an unaccomplished keratinocyte layer structure. Collectively, histological and immunofluorescent staining clearly demonstrated that the paracrine factors of triangular prism or cuboid patterned MSCs could facilitate wound healing by enhancing re-vascularization and re-epithelialization.

The promotion of skin repair by the paracrine products of specific patterned MSCs may be related to the increased VEGF, FGF and TGF proved in the cell experiments, which would facilitate vascularization of the wound and promote regeneration. Based on these new insights into how 3D micropatterns-mechanical signals influence the paracrine function of MSCs, our findings contribute to a better understanding of mechanical regulation mechanisms in the interaction of cells with their extracellular environment.

## CONCLUSIONS

In summary, 3D micropatterns triggered mechanical signals transduction and its influence on paracrine function of MSCs were investigated. We successfully fabricated 3D micropattern arrays with different curvature shapes (cylinder and triangle prism) and aspect ratios (cube and cuboid). We discovered that the 3D micropatterned cues were sensed by FAs and transduced through F-actin stress fibers to regulate nuclear tension. 3D micropatterns induced the orientation of F-actin and affected nucleus remodeling. Specifically, the ordered F-actin fibers were distributed over the whole nucleus in triangular prism and cuboid micropatterns, which increased tension in the nucleus, stretched nuclear pores and enhanced YAP nuclear import. More importantly, the activation of YAP in triangular prism and cuboid micropatterns significantly enhanced the paracrine function of MSCs, resulting in the enhancement of vascularization and wound remodeling in a rat model. The current study indicates that the topographical characteristics and 3D micropattern force of biomaterials can be well designed to regulate cell function and promote tissue regeneration by mechanotransduction pathways. Such knowledge is anticipated to play a vital role in the development of future cell/gene-active biomaterials.

## MATERIALS AND METHODS

Detailed materials and methods are available in the supplementary information.

## Supplementary Material

nwad165_Supplemental_File
