# Supplementary material for: 3D micropattern force triggers YAP nuclear entry by transport across nuclear pores and modulates stem cells paracrine
Source: Natl Sci Rev. 2023 Jun 1;10(8):nwad165. doi: 10.1093/nsr/nwad165 (PMC10347367; doi:10.1093/nsr/nwad165)
Supplement: nwad165_Supplemental_File [file nwad165_supplemental_file.pdf]

RESEARCH ARTICLE

MATERIALS SCIENCE

3D micropattern force triggers YAP nuclear entry by transport across nuclear pores and modulates stem cells paracrine

Yan Li[1,2,§], Zhenyu Zhong[1,2,§], Cunjing Xu[1,2], Xiaodan Wu[1,2], Jiaqi Li[1,2], Weiyong Tao[1,2], Jianglin Wang[1,2], Yingying Du[1,2,3,*] and Shengmin Zhang[1,2,3,*]

[1]Advanced Biomaterials and Tissue Engineering Center, Huazhong University of Science and Technology, Wuhan 430074, China;

[2]Department of Biomedical Engineering, Huazhong University of Science and Technology, Wuhan 430074, China;

[3]Research Base of Regulatory Science for Medical Devices, National Medical Products Administration & Institute of Regulatory Science for Medical Devices, Huazhong University of Science and Technology, Wuhan 430074, China

***Corresponding authors**. E-mails: yingyingdu@hust.edu.cn; smzhang@hust.edu.cn

§Equally contributed to this work.

Supplementary Information-Materials and Methods

Fabrication of 3D micropattern arrays: Micropatterns with different geometries were designed by AutoCAD produced on photomask by high-resolution laser printing. Through the standard photolithography, silicon mask with the devised micropatterns was produced by SU-8 (GM 1070)on silicon slice. The PDMS stamps were used to mold gelatin hydrogel 3D micropatterns. The PDMS molds were generated by pouring a mixture (10:1) of silicone elastomer base solution and curing agent (Sylgard 184) on the patterned silicon master. The Petri dish containing both the master with PDMS prepolymer was placed in an oven at 70 °C and cured for 6 h. The as-prepared PDMS with a microwell pattern was separated from the silicon wafer and used in the following assay. The gelatin solution (10%, w/v) was coated on the PDMS molds. After fixed in the ice-cold glutaraldehyde solution (1%, v/v) for 1 h for crosslinking, the 3D micropatterned gelatin was thoroughly washed with the distilled water and fixed with NHS-PEG-OH (1%, w/v) for 2 h. In order to enhance cell adhesion and spreading, the inside of 3D micropatterns was functionalized with fibronectin. Subsequently, the hydrogel with 3D micropatterns was imaged via the confocal microscopy. Images were reconstructed from Z-stacks and dimensions were analyzed using Fiji software.

MSCs culture in 3D micrpatterns: The MSCs were obtained from Cyagen Inc and cultured in DMEM low glucose (Gibco) supplemented with 10% FBS (Gibco), 1% L-glutamine, and 1% Pen/Strep (Thermo fisher scientific) at 37 °C with 5% CO_2. The fourth to sixth passages of MSCs were used in this study. Cells were seeded in the 3D microwells at a certain density and incubated

for 10-15 min. The gelatin hydrogel was subsequently washed with a gentle flow of medium for several times to remove the nonadherent cells.

Cell staining: The MSCs were fixed with 4% paraformaldehyde (Sigma) for 30 min after 3 days of culture in 3D micropatterns, followed by washing three times with PBS and then permeabilized with 0.3% Triton X-100 (Sigma), washed three times with PBS, and incubated with 2% bovine serum albumin (BSA) in PBS for 2h. Subsequently, the cells were incubated with primary antibodies overnight at 4 °C. The cells were stained for vinculin (Sigma, 1:1000 dilution), mysoin (Abcam, 1:500 dilution), Lamin A/C (Cell signaling, 1:1000 dilution), YAP (Cell signaling, 1:1000 dilution). MSCs were washed with PBS three times and then incubated with the appropriate secondary antibodies (Guge, 1:200 dilution) for 2h at room temperature. Cell nuclei was stained with DAPI (Invitrogen, 1:1000 dilution). F-actin was stained with Phalloidin 488 (in MSCs) or Phalloidin546 (in HUVECS) (Invitrogen, 1:1000 dilution). Images were taken using confocal laser scanning microscope (Olympus, Japan). Z-stacks were recorded at either 1 μm (vinculin, Lamin A/C) or 0.5 μm (F-actin, mysoin, YAP and nucleus), z-spacing at 1024 × 1024 pixels. Laser power and detector settings were kept constant during the imaging of the different groups.

Microscopy data analysis: All confocal pictures were taken with different z-stacks and overlaid in Fiji software with plugin. To quantify fluorescence intensity, images were taken by confocal microscope with identical camera setting. For generating heat maps of F-actin staining, raw fluorescent images were aligned in Fiji with the same orientation, incorporated into a Z stack and the average intensity calculated for heatmap generation. For the F-actin analysis, we calculated the orientation order parameter (OOP) for F-actin at each slice, using the equation $\sqrt{(\sin 2\theta_i)^2 + (\cos 2\theta_i)^2}$, where θ is the fiber orientation at each pixel. The OOP which increases from 0 for randomly oriented features up to 1 for perfectly aligned features. To quantify the variation in nuclear shape, we utilized Fiji software to extract the contours of individual stained nucleus, determining the length axis, short axis, perimeter, and projected area of each nucleus. The nuclear shape factor (NSF) was calculated from the projected area and perimeter of the nucleus using the formula: $NSF=4\pi \times area/(perimeter)^2$. The assumed value of NSF is between 1 (circular shape) and 0 (elongated shape) [1]. To assess the spatial chromatin condensation, we employed a quantitative approach based on DAPI staining. The integrated fluorescence intensity was calculated as the sum of the pixel intensities. The integrated fluorescence intensity was then divided by the nuclear volume to obtain the average spatial density, which was related to the average chromatin packing ratio, and was indicative of chromatin condensation [2].

3D imaging with Imaris: Fuorescence imaging was obtained using confocal laser scanning microscope to obtain 3D reconstructive images of the cell morphology (F-actin staining with phalloidin) and nucleus morphology (DAPI staining). Each sample was imaged using the multiple z-stack images with each step of 0.5 μm. The 3D reconstruction of cell was performed using Imaris software as previously descried [3].

Transmission electron microscopy (TEM): The MSCs were seeded in 3D micropatterns for 7 days and fixed with 2,5% glutaraldehyde for 1 h at room temperature. The cell pellets were embedded with 1% agarose solution before being immobilized with 1% osmic acid solution for 2 h at room

temperature. Then, dehydration in an ethyl alcohol series (30%, 50%, 70%, 80%, 95%, 100%, 20 min each) and dehydration in 100% acetone, 15 min. The embedding models were infiltrated and embedded in resin. Ultrathin sections with a thickness of 60-80nm were obtained using an ultramicrotome (Leica UC7, Leica). Sections were observed in TEM (HT7800, HITACHI). In images, the nuclear pores observed with the visible nuclear basket were selected in the cross-section. The nuclear pore length was measured from one side of the double bilayer, at the point where the nuclear basket (black line) begins, to the other side of the double bilayer where the nuclear basket ends.

Gene chip analysis: The MSCs were cultured in 3D micropatterns for 7 days, and then the cells were collected to perform the Gene chip analysis with the help of Wuhan Bohao Tech Co., Ltd. The gene transcriptome date was analyzed using the Gene Ontology Database.

Gene expression analysis: The MSCs were cultured in 3D micropatterns for 7 days. Then, the total RNA was harvested with a Total RNA Kit (HP Total RNA Kit, R6812-02, OMEGA, USA). The gene expression analysis of paracrin factors VEGF, TGF, FGF was performed via the quantitative real-time reverse transcription-polymerase chain reaction (RT-PCR). The $2^{\Delta\Delta C}$ method was applied for evaluating the gene expression level, compared with housekeeping gene GAPDH.

Enzyme-Linked ImmunoSorbent Assay (ELISA) measurements: For paracrine profile measurement, the conditioned medium (CM) for each group was collected based on previously reported protocols [4]. ELISA measurements for pro-regenerative cytokines VEGF, FGF, TGF, in CM were conducted according to the manufacturer's instructions (R&D Systems, USA).

Inhibition assays: The YAP inhibitor (Verteporfin) was used at a concentration of 5 μM, and the F-actin inhibitor (Cytochalasin D) was used at a concentration of 0.2 μM. The size of nuclear pores and YAP nuclear localization in MSCs cultured in 3D micropatterns with F-actin inhibitor co-incubation were detected. Protein secretion level of paracrine factors VEGF, FGF, TGF in MSCs cultured in 3D micropatterns with YAP inhibitor addition was measured via ELISA.

In vitro tube formation assay: The pro-angiogenic potential of paracrine factors contained in micropatterned MSCs-conditional medium (CM) was tested by the tube formation assay of HUVEC. Briefly, Matrigel (BD, USA) was added to pre-cooled 96-well plates at 50 μL/well. After a 1-hour-solidification process at 37℃, The HUVECs of 5×10^4 were resuspended in 100 μL of different CM and seeded into each well. The 96-well plate was incubated in a 5%CO2-incubator at 37℃ for another 6 hours. Then the HUVECs were fixed with 4% paraformaldehyde for 20 min and stained with Phalloidin 546 (Invitrogen) for F-actin. The tube formation was observed with a fluorescence microscope (OLYMPUS) and the number of tubes was calculated using Fiji software.

Wound simulation scratch assays: The pro-migratory potential of paracrine factors contained in CM was tested by the wound simulation scratch assay of NIH-3T3. A linear scratch was scraped by a sterile 200 μL pipette tip and the drifting cells were removed by PBS. Then the different CM was added, the closure of the scratch was captured by fluorescence microscope (Nikon) after the culture of 24 h.

***In vivo* wound healing experiments:** Animal protocols were approved by the Experimental Animal Ethics Committee of Huazhong University of Science and Technology (approval number: [2018] IEC (S313)). 45 Sprague Dawley (SD) female rats (about 180 g) were purchased from Hubei Provincial Center for Disease Control and Prevention (HBCDC), China. The rats were individually anesthetized by breathing with the inhaled isoflurane (2–3% isoflurane, 2 L min−1 oxygen). Four full-thickness excisional skin wounds (diameter: 13 mm) were created on the dorsum of each rat. The rats were divided into five groups, the gauze cover was set as the blank control. After stitching up the skin incision, 20000 UI penicillin was injected to each rat intramuscularly. To evaluate the wound contraction rate, a digital camera (EOS 1300D, Canon, Japan) was applied to capture the images of wound beds during the whole healing process. The wound areas of each wound at different time intervals were analyzed using Fiji software. The wound contraction rate was calculated using the following equation

Wound contraction rate [%] = (A0 – At) / A0 × 100%

where A0 and At referred to the initial wound area and the wound area at predeterminate time intervals, respectively.

Histological, Immunohistochemical, and Immunofluorescent Analysis: The regenerated skin tissues were harvested at 3,7 and 14 days postoperatively. The 4% paraformaldehyde fixed skin samples were embedded with paraffin. All the samples were sliced into 4 μm sections for staining analysis. The wound healing effect was evaluated by hematoxylin-eosin (HE) staining. Immunofluorescence (IF) assays of CD31 (marker of newly formed blood vessels), CK10 (marker of keratinocytes) were also conducted to give a comprehensive assessment of vascularized skin regeneration.

Statistical analyses

Statistical analysis was performed with Origin software and quantitative results were expressed as mean ± s.d. Statistical analyses were carried out using unpaired, two-sided Student's t-test. *$p < 0.05$ was considered significant, **$p < 0.01$ was considered highly significant, ***$p < 0.001$ was considered very highly significant.

Supplementary Figures

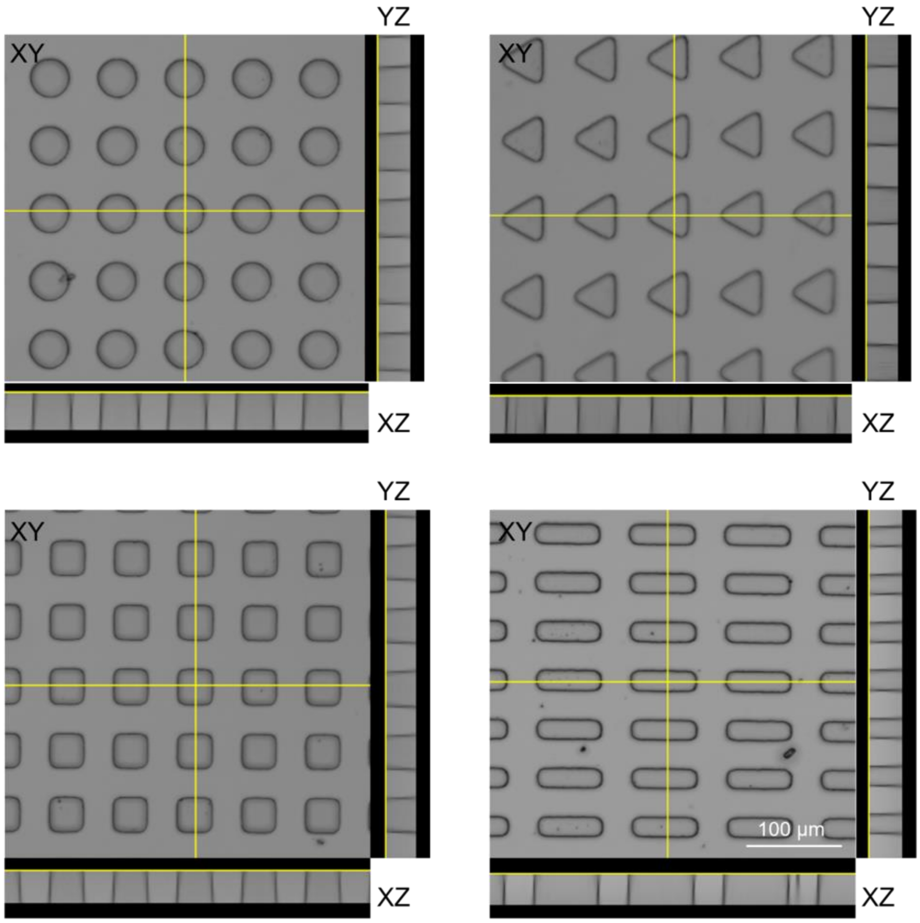

Fig. S1. Confocal image shows gelatin hydrogel containing microwells of various geometries. Here the 3D micropattern cross-sections were imaged in the x-z (bottom) and y-z (right) planes.

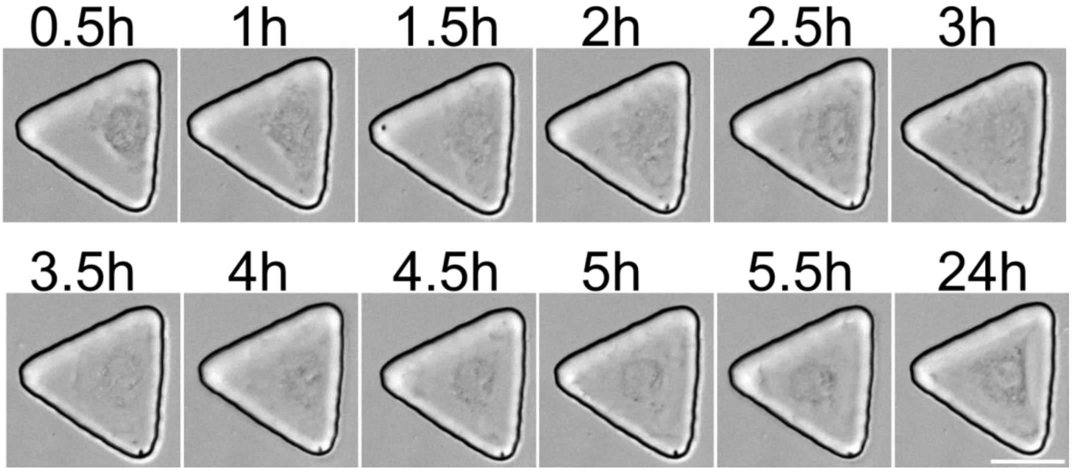

Fig. S2. Live imaging showing a representative MSCs spreading in a triangular prism niche within 24 hours after encapsulation. Live cell imaging of MSCs in 3D micropattern revealed that the cells spread rapidly after encapsulation. Scale bar, 20 μm.

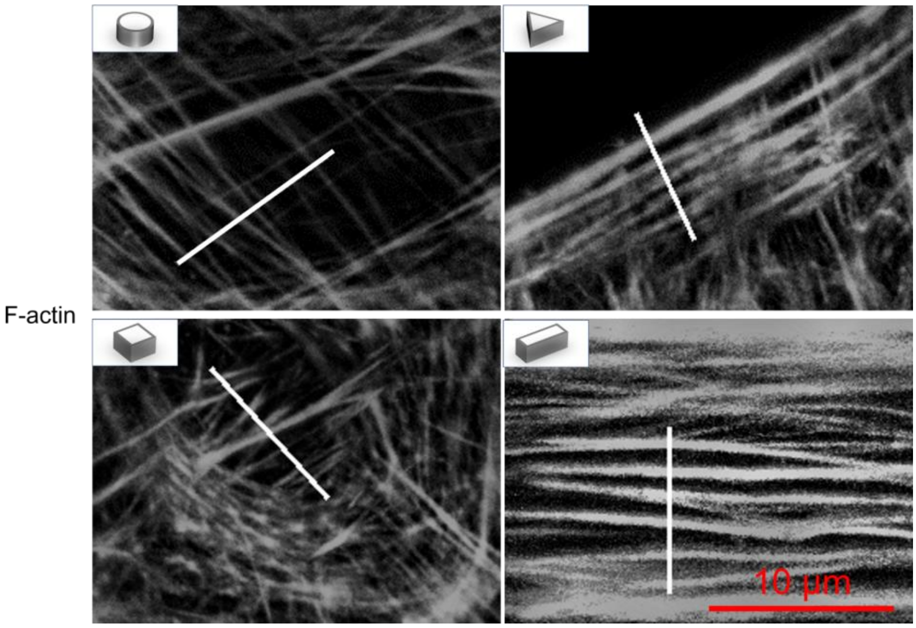

Fig. S3. Analysis of the thickness of F-actin fibers. Intensity plot of the actin fibers (Fig. 2 e and f) was processed along the white lines. Scale bar, 10 μm.

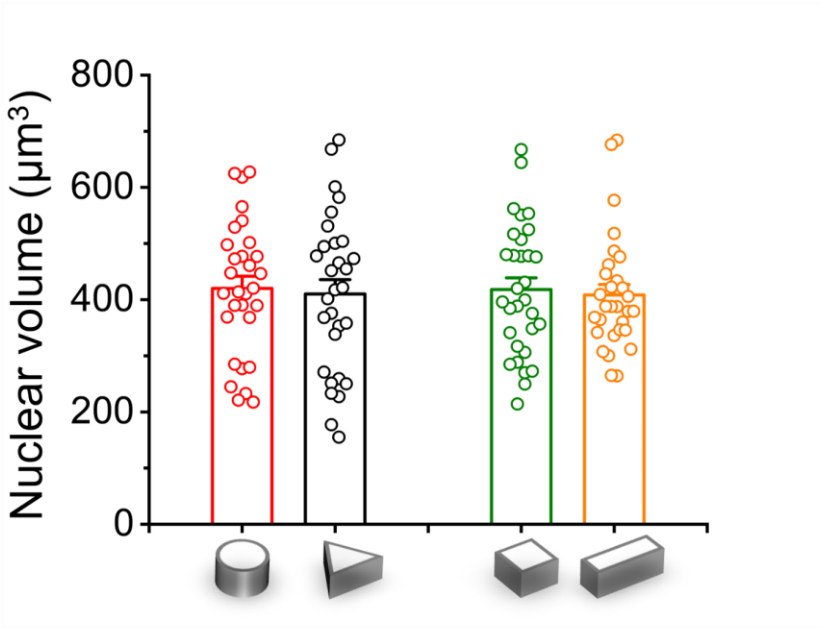

Fig. S4. Quantification of nuclear volume of MSCs cultured in 3D micropatterns; n = 30-40 cells per condition.

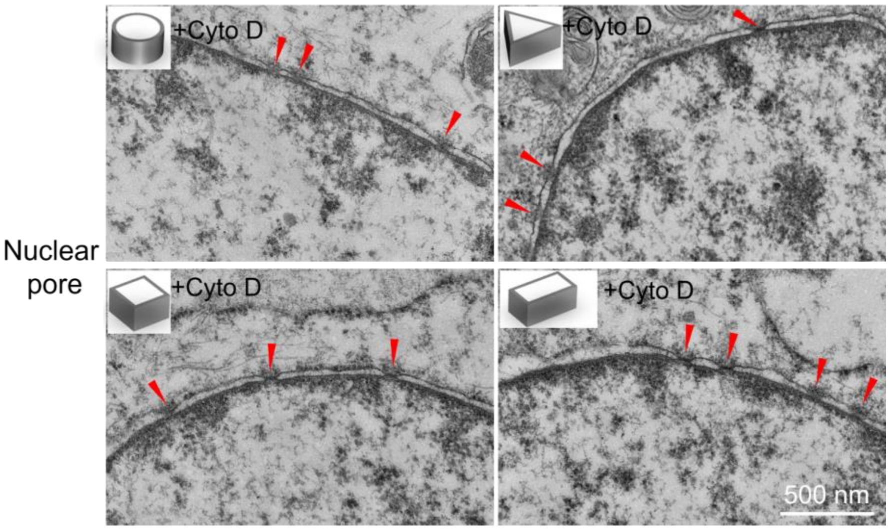

Fig. S5. TEM images of nucleus pores in 3D micropatterned cells after treatment with 10 μM Cytochalasin D (Cyto D). Scale bar, 500 nm. Inhibitor effects of F-actin on nuclear pore size in 3D micropatterned MSCs.

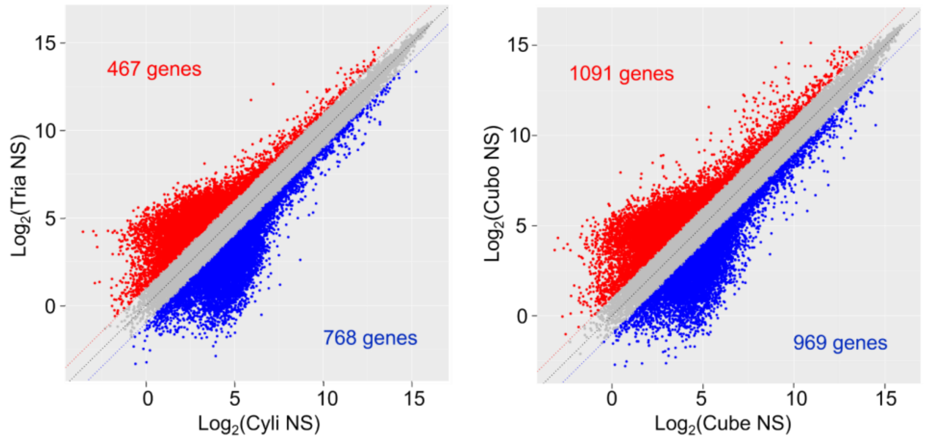

Fig. S6. a Differential gene expression in triangular prism (Tria) group comparison to cylinder (Cyli) group using gene chip. Genes with upregulation (red) or downregulation (blue) are shown in colour. b Differential gene expression in cuboid (Cubo) group comparison to cube group using gene chip. Genes with upregulation (red) or downregulation (blue) are shown in colour.

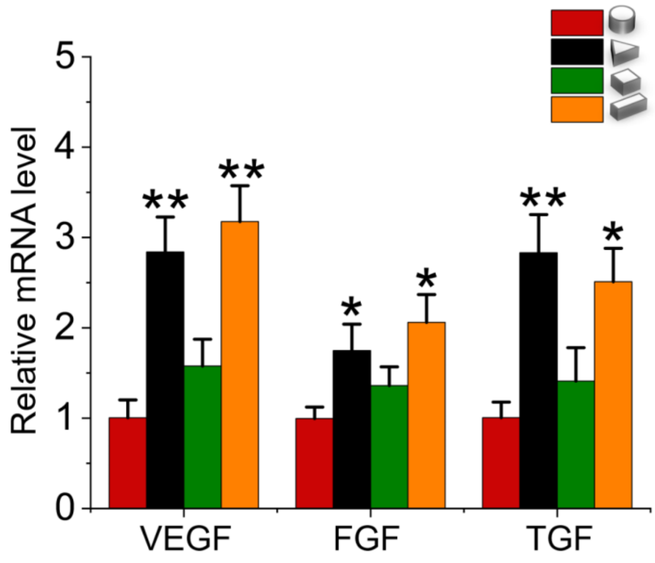

Fig. S7. Relative mRNA expression of the angiogenesis-related and tissue repair-related factors in MSCs cultured in 3D micropatterns via RT-PCR analysis. The data are represented as the mean ± SD, * $p<0.05$, ** $p<0.01$.

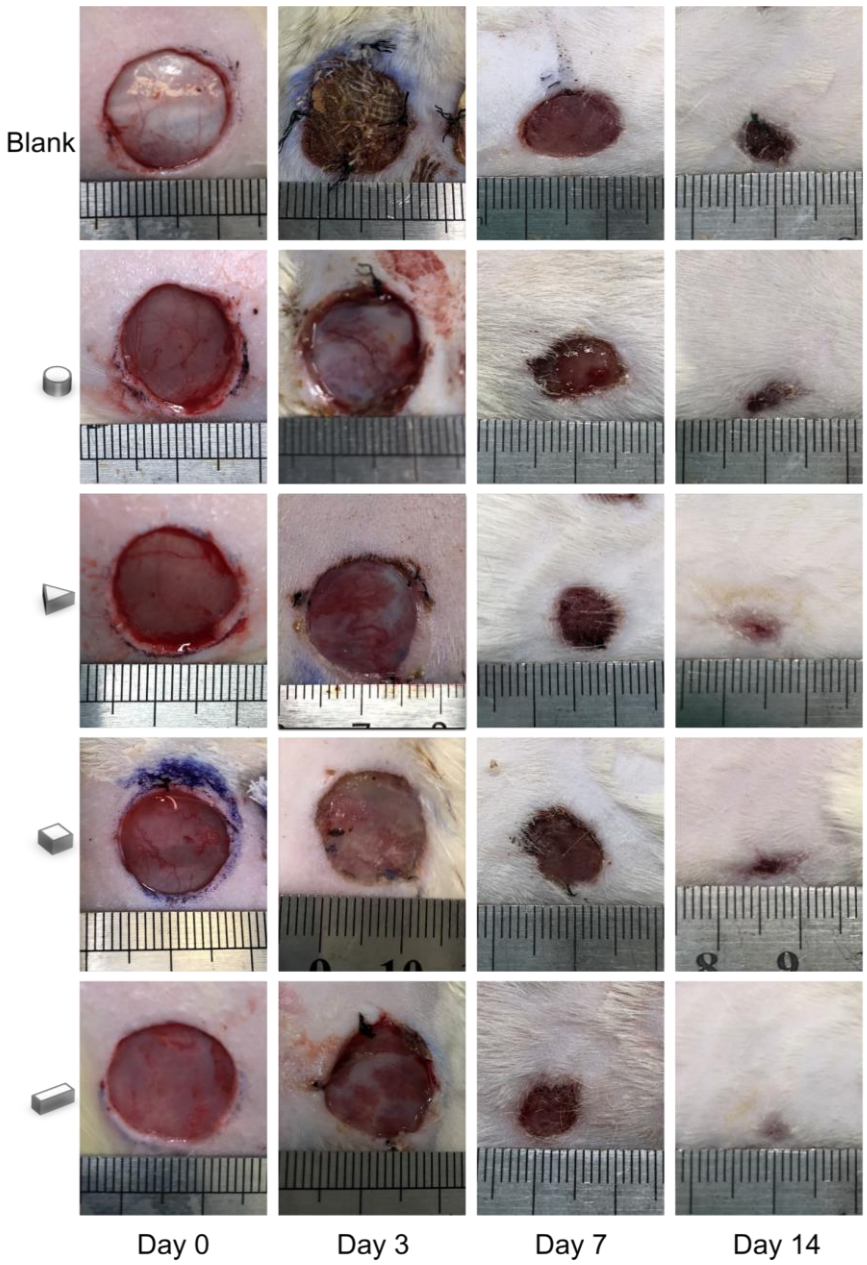

Fig. S8. Photographs of full-thickness cutaneous wounds on day 0, 3, 7 and14.

References

1. Kim JK, Louhghalam A and Lee G *et al.* Nuclear lamin A/C harnesses the perinuclear apical actin cables to protect nuclear morphology. *Nat Commun* 2017; **8**: 2123.

2. Jain N, Iyer KV and Kumar A *et al.* Cell geometric constraints induce modular gene-expression patterns via redistribution of HDAC3 regulated by actomyosin contractility. *Proc Natl Acad Sci U S A* 2013; **110**: 11349-54.

3. Hou Y, Xie W and Yu L *et al.* Surface roughness gradients reveal topography-specific mechanosensitive responses in human mesenchymal stem cells. *Small* 2020; **16**: e1905422.

4. Lian M, Sun B and Han Y *et al.* A low-temperature-printed hierarchical porous sponge-like scaffold

that promotes cell-material interaction and modulates paracrine activity of MSCs for vascularized bone regeneration. *Biomaterials* 2021; **274**: 120841.
